# Supplementary material for: A pan-cancer analysis of homeobox family: expression characteristics and latent significance in prognosis and immune microenvironment
Source: Front Oncol. 2025 Feb 6;15:1521652. doi: 10.3389/fonc.2025.1521652 (PMC11840236; doi:10.3389/fonc.2025.1521652)
Supplement: Supplementary Figure 1 — Mutations of each HOX gene in 32 cancers (TCGA, PanCancer Atlas) by cBioportal. [file DataSheet1.zip › Suppl.files/raw code.zip!/code/4.survival/survivalplot.zip!/survivalplot/HOXD4_OS.ESCA.pdf]

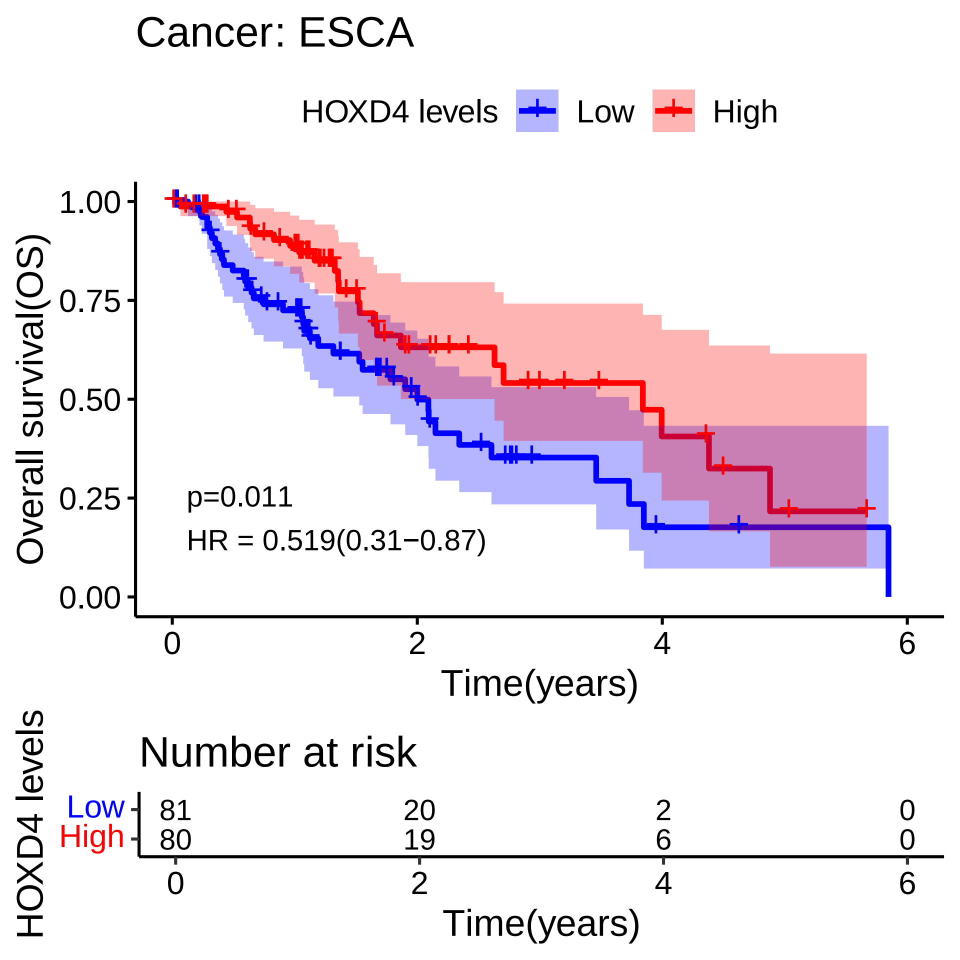
Cancer: ESCA
HOXD4 levels
Low
High
Overall survival(OS)
1.00
0.75
0.50
0.25
0.00
p=0.011
HR = 0.519(0.31−0.87)
0
2
4
6
Time(years)
Number at risk
HOXD4 levels
Low
High
81
20
2
0
80
19
6
0
0
2
4
6
Time(years)
